# Device-worn measures of sedentary time and physical activity in South Asian adults at high risk for type 2 diabetes in Metro-Vancouver, Canada

**DOI:** 10.1371/journal.pone.0266599

**Published:** 2022-05-05

**Authors:** Bushra Mahmood, Lindsay Nettlefold, Maureen C. Ashe, Joseph H. Puyat, Tricia S. Tang

**Affiliations:** 1 Faculty of Medicine University of British Columbia, Vancouver, British Columbia, Canada; 2 Centre for Hip Health and Mobility, University of British Columbia, Vancouver, Canada; 3 Department of Family Practice, University of British Columbia, Vancouver, British Columbia, Canada; 4 Centre for Health Evaluation & Outcome Sciences, Saint Paul’s Hospital, Vancouver, British Columbia, Canada; 5 School of Population and Public Health, University of British Columbia Vancouver, British Columbia, Canada; Universiti Malaya, MALAYSIA

## Abstract

**Background:**

South Asians have high incidence of chronic disease. Physical activity (PA) and sedentary time are modifiable risk factors for chronic disease but their assessment in South Asians has been primarily based on self-report. This study presents directly-measured PA and sedentary time in South Asian adults in Canada.

**Methods:**

A subset of 100 South Asian participants from a larger study who were identified at being at a higher risk for type 2 diabetes wore Actical accelerometers for 7 days. Anthropometric measures were taken and socio-demographic factors including age, income, education level, years since immigration, presence of children under the age of 12 years in the household and employment status were self-reported.

**Results:**

Ninety-one participants (mean age 65.6 years) provided valid accelerometer data. Participants accumulated mean 673.5 (95% CI: 656.6, 691.0) min/day sedentary time, 130.5 (95% CI: 117.3, 145.3) min/day light PA (LPA) and 2.3 (95% CI: 1.3, 4.2) min/day moderate-to-vigorous PA (MVPA). For sedentary time and LPA, sex and BMI explained 51% of variability. For MVPA, BMI, season of assessment and employment status explained 23% variability with those who were employed accumulating significantly higher mean min/day of MVPA compared to those who were unemployed; (5.8, 95% CI: 1.5, 21.7) vs (1.5, 95% CI: 5.3, 20.0) respectively.

**Conclusion:**

High sedentary time, and low MVPA indicates the need to focus health promotion efforts on shifting sedentary time into LPA while trying to increase MVPA. Future studies need to be based on larger, representative samples of South Asians.

## Introduction

Regular physical activity (PA) decreases the risk of chronic diseases like cardiovascular disease, hypertension, colon cancer and type 2 diabetes [1, 2]. In contrast, prolonged sedentary time is associated with a higher risk of developing metabolic syndrome [3]. Sedentary time is now recognized as not just the absence of PA but as a distinct set of behaviors with unique health outcomes independent of those associated with a lack of leisure time PA [4–6]. Thus, an accurate assessment of sedentary time in a population is equally important as that of accurate levels of PA.

Individuals of South Asian descent (i.e. from India, Pakistan, Bangladesh, Nepal, Bhutan, Sri Lanka or Maldives) living in western countries are at a higher risk of developing cardiovascular disease, hypertension, type 2 diabetes mellitus and dyslipidemia at younger ages and lower Body Mass Index (BMI) compared to White individuals [7–9]. Despite the evidence-based health benefits, PA levels among South Asians remain low compared to the general population [10, 11]. For instance, South Asians living in England were 60% less likely than White individuals to meet recommendations of 150 minutes of moderate to vigorous PA (MVPA) per week [11]. In Canada, only 34% of South Asians were moderately active (≥1.5 kcal⋅kg^-1^⋅day^-1^) lowest among seven other ethnic groups, with the lowest prevalence of moderate activity (12%) observed in South Asian women [12]. Similarly, in the United States (US), the prevalence of physical inactivity was close to 60% in South Asian immigrants [10].

Most population-based studies on PA have employed self-report for measuring sedentary time and PA as well as compliance with meeting PA guidelines of 150 min/week of MVPA [13]. Thus, much of our information regarding levels of PA among South Asians in Canada is also based on self-report which consistently shows South Asians being one of the least active groups when compared with other ethnic groups [12]. Self-report is subject to inherent limitations like social desirability bias and imperfect recall as well as difficulty in assessing accurate frequency, duration and intensity of activities [14]. In one study comparing sedentary time and PA among South Asian and White participants with both direct assessment as well as self-report, correlation coefficients between self-reported and directly measured PA stratified by ethnic group ranged from weak to moderate (ρ = 0.17–0.50), tending to be stronger in South Asian participants compared to White participants [15]. In another study with a male South Asian sample (mean age 45 years), International Physical Activity Questionnaire-Short Form (IPAQ-SF) derived MVPA was significantly lower than accelerometer-derived MVPA (p < 0.001). IPAQ-SF derived sedentary time was significantly higher than accelerometer-derived sedentary time (p < 0.001) [16]. Curry et.al, in her sample of South Asian women found correlations were not significant between accelerometer and IPAQ-SF-assessed MVPA (r = −.12, p = .58) and sedentary time (r = −.14, p = .23) concluding that the IPAQ-SF may not accurately measure PA in South Asian women in the United Kingdom (UK) as shown by qualitative evidence indicating several issues with interpretation and recall of PA and sedentary time as assessed via this questionnaire [17]. Considering the substantially higher risk of cardiovascular disease and metabolic syndrome among South Asians and the role of a physically active lifestyle in mitigating this risk, more research based on accelerometers to directly assess PA and sedentary time and use these to validate self-report tools is needed. Accurate assessment is imperative to track changes in PA and sedentary time and also to develop targeted health promotion programs and to evaluate the effectiveness of interventions designed to increase activity levels and/or decrease sedentary time [14].

Accelerometers are easy to wear, small motion sensors that can record frequency, duration and intensity of PA, as well as sedentary time, in a free living environment [18]. Accelerometers are particularly useful to measure PA and sedentary time in diverse ethnic groups where English proficiency may be limited and where conceptualization of PA and sedentary time may be heavily influenced by cultural factors [11, 19]. While there has been a rapid increase in the use of accelerometers in the past decade, our recent systematic review concluded that up until March 2019, just 14 studies (with only three of those based in North America) used this technology to measure PA levels and/or sedentary time in South Asians [20].

For public health surveillance and development of effective interventions, it is important to monitor not only the levels and trends of PA and sedentary time but also to investigate socio-demographic correlates of PA and sedentary time so that health practitioners can identify some of the modifying factors [21] and develop targeted interventions. Previous studies have observed associations between PA and socio-demographic variables such as sex, age, education level, marital status, the presence of young children in the home, immigration status, and household income [22–24] but few studies have investigated these potential associations in South Asians. Curry et al. observed an association between age and waist circumference where these variables explained 7.3% and 14.9% of the variability in LPA and MVPA respectively in South Asian women in UK.

Thus, the aims of this cross-sectional study are to 1) report the overall levels and patterns of device-measured PA and sedentary time among South Asian adults in Metro Vancouver, British Columbia, Canada; and 2) examine potential socio-demographic correlates of PA and sedentary time in this population.

## Methods

### Study population

We recruited a subset of 100 men and women from a larger study of 425 South Asians. Eligibility criteria for the larger cohort included being a South Asian adult (≥21 years of age) living in Metro Vancouver, Canada, with no previous diagnosis of diabetes; being able to speak Punjabi and/or English and observed to be at an increased risk of diabetes based on the 7-item American Diabetes Association diabetes risk test. Recruitment for the larger study took place between July 2013 and June 2014 from Sikh Gurdwaras and Hindu temples in Metro Vancouver, Canada. Further information on recruitment has been provided elsewhere [25]. All participants provided written informed consent before participation in the study.

The ethics review committee of the University of British Columbia (H13-00189) and Fraser Health Research Ethics Board (FHREB 2013–030) approved this study.

### Measures

#### Socio-demographic data

Participants self-reported socio-demographic data including age, sex, marital status, religion, country of birth, years since immigration to Canada, highest education level, employment status, total household income, and presence of children under 12 years of age in the household.

#### Anthropometric measures

We assessed weight (kg) and height (m) with a digital weighing scale (Seca 874) and a stadiometer (Seca 213), (Seca, CA, USA) and calculated BMI as kg/m^2^. BMI was classified as healthy if it ranged between 18.5–24.9 kg/m^2^, over-weight if between 25–29.9 kg/m^2^ and obese if ≥30 kg/m^2^ [26].

We measured waist circumference (WC) using a flexible Seca waist tape at the level of the umbilicus. For women, WC was classified as healthy if <35 inches and unhealthy if ≥35 inches. For men, WC was considered healthy if < 40 inches and unhealthy if ≥40 inches [26].

### Device-worn measures of physical activity and sedentary time

We assessed step count, PA, and sedentary time with Actical accelerometers (Philips Respironics, Oregon, US). The Actical (dimensions: 2.8 x 2.7 x 1.0 centimeters; weight: 17 grams) measures and records time-stamped acceleration in all directions [6] and has been validated to measure PA in adults [27]. Wearable devices containing triaxial accelerometers, such as GENEA, GENEActive, Actigraph, and RT3, measure acceleration in 3 orthogonal axes whereas omnidirectional accelerometers such as MiniMitter and Actical assess acceleration in multiple directions but are most sensitive to movement in the vertical plane [28]. A study investigating the validity of the Actical activity monitor for assessing steps and energy expenditure during walking concluded that the step count function of the Actical accelerometer provides valid estimates of step counts at 83 and 133 m^.^min^(-1)^ on a range of healthy participants [29]. A review of the usefulness of motion sensors to estimate energy expenditure in children and adults found that devices that presented the highest validity compared with Doubly Labelled Water were the Actical and ActiReg, and concluded that adding biometric information seemed to be an advantage to estimate adults’ Total Energy Expenditure [30]. Additionally, it found that accelerometers were most accurate when placed on the hip (compared with wrist placement), were most accurate for detecting sedentary behavior, and were least accurate for detecting light activity [31].

Participants wore the monitor on an elasticized belt over their right hip for seven consecutive days and were asked to take it off only during water related activities.

### Data reduction and statistical analysis

We downloaded accelerometer data using Actical data analysis software (Philips Respironics, Oregon, US). We processed our data using Kinesoft software (KineSoft, Loughborough, UK). To be included in the analysis, participants had to have a minimum of three days of data with a valid day defined as ≥10 hours (600 minutes) of monitor wear time [6]. Non-wear time was defined as at least 60 consecutive minutes of zero counts, with allowance for 1 to 2 minutes of counts between 0 and 100 [6]. Wear time was defined by subtracting non-wear time from 24 hours. Epoch length was set at 60 seconds based on previous research [32]. Anything above 20,000 counts per minute (cpm) was considered biologically implausible and therefore treated as spurious data and deleted [33].

We applied the step count categories and classification system of Tudor-Locke and Bassett to classify our participants with <5000 mean steps/day as sedentary, 5000–7499 steps/day as physically inactive, 7500–9999 steps/day as moderately active, ≥10,000 steps/day as physically active, and ≥12,500 as very active [34].

We used recommended cut points to calculate time (min/day) in the following intensity categories: sedentary time (<100 cpm), light PA (LPA) (100 − < 1535 cpm), moderate PA (MPA) (1535 to < 3962 cpm), vigorous PA (VPA) (≥3962 cpm) and moderate to vigorous PA (MVPA) (≥ 1535 cpm) as in previous research [35]. We defined adherence to PA guidelines as a weekly sum of 150 or more minutes of MVPA per week. If respondents had three to six valid days, their average daily MVPA was multiplied by seven to obtain a weekly sum [6].

We classified time of day as morning (from 6:00 am till 11:59 am), afternoon (12:00 pm till 5:59 pm) and evening (6:00 pm till 11:59 pm). Since recruitment for the accelerometer trial took place at different times of the year, we also investigated any potential seasonal effects on PA and sedentary time. Seasons were classified as: spring (March 20 –June 20), summer (June 21^st^–Sept 22^nd^) fall (Sept 23^rd^–Dec 21^st^) and winter (Dec 22^nd^–March 19^th^) [36].

We conducted descriptive analyses (mean, standard deviation, and/or median, Inter Quartile Range (IQR) and percentage as appropriate) for all variables. We assessed differences in socio-demographic characteristics between the larger study cohort and the accelerometer subset using independent t-test for numerical data (or Wilcoxon for data not normally distributed) and Chi-square for categorical variables (or Fisher’s Exact test where the expected count in cells was less than 5). As PA and sedentary time data were not normally distributed, we log transformed data for analysis and exponentiated results to transform back to the original scale.

We used Independent t-tests or one-way Analysis of variance (ANOVA) with paired Tukey HSD tests to determine differences in PA and sedentary time between levels of the socio-demographic variables (e.g., age, sex, BMI, religion, years since immigration, income, education, seasons and weekdays/weekend) [37]. We also checked for correlation between our three primary outcomes: sedentary time, LPA and MVPA using Pearson correlation. To explore patterns of PA and sedentary time by sex throughout the day (morning, afternoon, evening), we used repeated measures ANOVA.

We assessed any potential association of the socio-demographic variables on sedentary time, LPA and MVPA using multiple linear regression. We calculated Pearson’s correlation coefficients to check for any potential collinearity between the independent variables. An a priori list of variables to be added into the models was created based on literature review of similar studies. These variables were age, sex, BMI, waist circumference, marital status, years since immigration, education, income, job status. As some studies have observed significantly different clinical outcomes associated with religion among various South Asian sub-groups, we also investigated potential association if any, of religion on our study outcomes [11, 38]. Life changing events such as marriage and having young children in the household have been known to impact lifestyle behaviors like sedentary time and PA [17, 39, 40]. Therefore, we explored both marital status and presence of children under 12 years of age in the household on sedentary time, LPA and MVPA. Each potential confounder’s effect on model fit (adjusted R^2^, P-value) was observed—the only variables that were kept were those that were not correlated, and had a significant effect or improved model fit. Based on our knowledge of previous literature on correlates of PA in ethnic populations, we also evaluated known interactions of various variables such as age, sex, BMI, income, education, years since immigration, season and job status with our outcome measures (sedentary time, LPA and MVPA). All statistical analyses were conducted using SAS (SAS Institute, Cary, NC) version 9.4.

## Results

### Participant characteristics

Demographic and questionnaire data for 425 participants (n = 325 full study cohort; n = 100 accelerometer subset) are shown in Table 1.

**Table 1:** Socio-demographic characteristics of participants in the parent study (n = 425), the non-accelerometer sample (n = 327) and the accelerometer sub-study (n = 100).

	Overall			No Accelerometer			Accelerometer			p Value
	(N = 425)			(N = 325)			(N = 100)			
	N	%	Mean (SD)/ Median (IQR)	N	%	Mean (SD)/ Median (IQR)	N	%	Mean (SD)/ Median (IQR)	
**Age (mean (SD)**	425	100	65.3 (10.5)	325	100	65.2 (10.7)	100	100	65.6 (10.8)	0.87
**Age Categories (Years)**										
20–59	109	26		85	26		24	24		0.4
60–79	278	65		208	64		70	70		
80>	38	9		32	10		6	6		
**Sex**										0.7
Women	177	42		134	41		43	43		
Men	248	58		191	59		57	57		
**BMI (kg/m2) (mean, SD)**			28.1 (4.0)			28.2 (4.0)			28.0(4.6)	0.77
**BMI Categories (kg/m2)**										
18.5–24.9	78	18		60	18		18	18		0.8
25–29.9	235	56		181	56		54	54		
≥30	110	26		82	25		28	28		
**Waist Circumference (inches) (Mean, SD)**			39.7 (3.9)			39.6(4.0)			39.9 (3.6)	0.97
**WC Categories Women**										
Healthy (<35 inch)	11	10		6	11		5	8		0.1
Unhealthy (= >35 inch)	236	90		184	89		52	93		
**WC Categories Men**										
Healthy (<40 inch)	99	40		76	39		23	54		
Unhealthy (= >40 inch)	76	60		56	61		20	47		
**Country of Birth**										
India	397	95		310	95		90	92		0.17
Other	28	5		15	5		10	10		
**Years lived in Canada (median/IQR)**			21 (28)			20 (29)			26 (26.5)	0.1
**Years lived in Canada (Categories)**										
< 10	90	22		74	24		16	17		0.15
> = 10	318	78		238	76		80	83		
**Marital Status**										
Married	364	86		273	84		91*	93		0.03
Other	59	14		52	16		7	7		
**Language**										
Punjabi	400	95		309	95		91	93		0.4
English	252	60		190	58		62	63		
**Religion**										
Sikh	379	90		291	90		88	90		0.94
Other	44	10		34	10		10	10		
**Education**										
< High School	204	49		164	51		40	41		0.22
High School	119	28		87	23		26	27		
> High School	98	23		72	27		32	33		
**Employment**										
Currently working	114	27		90	28		24	24		0.52
Not working	308	73		234	72		74	76		
**Income (CAD $)**										
<20,000	149	41		75	28		22	25		0.57
20,000–49,999	97	27		108	40		41	46		
> = 50,000	115	32		89	33		26	29		
**Smoking History**										
Ever smoked/smoking	9	2		8	2		1	1		0.18
Never smoked	413	98		316	98		97	99		
**Children under 12**										
0	258	63		195	62		63	66		
1 or more	152	37		119	38		33	34		0.53

The mean age of the subset was 65.6 (10.8) years with 82% classified as over-weight/obese by BMI. With the exception of marital status (93% vs 84%, p = 0.03, for subset and the full study respectively), there were no statistically significant differences between participants in the full study and the subset. Of the 100 participants who participated in the accelerometer sub-study, nine had insufficient (<3 valid days) accelerometer data and were excluded from analyses.

A total of 91 participants (40 women) with complete demographic and anthropometric information and valid accelerometer data were included in the final sample for analysis.

### Accelerometer measured sedentary time and physical activity levels

Participants spent 11.2 hours/day (62.2% of the wake time) being sedentary and 2.2 hours/day (12% of the wake time) in LPA. On average, women accumulated significantly more sedentary time compared to men [694.6 vs 657.4 min/day (95% CI: 671.8, 718.3),]. While not statistically significant, there was a notable absolute difference of 60 min of sedentary time between the youngest (20–59 years) and the oldest (≥ 80 years) age group; (688.8 vs 628.1 min/day respectively). Similarly, married participants accumulated 58.7 min/day more in sedentary time than the ‘other’ category. LPA was significantly higher in the younger age group (20–59 vs ≥ 60) [186.1 vs 120.3 min/day (95% CI: 136.9, 206.4)], those with a BMI in the over-weight (25–29.9 kg/m^2^) vs healthy range (18.5–24.9 kg/m^2^), [142.9 vs 100.3 min/day (95% CI: 72.9, 130.8], married, [136 vs 81.7 min/day (95% CI: 121.7, 151.9)], and with annual income between $20,000–$49,999 vs <$20,000 [139.0 vs 96.5 min/day (95% CI: 117.9, 163.9)]. MVPA was significantly higher during the summer season [5.4 (95% CI: 2.4, 12.2)] vs winter [0.8 min/day (95%CI: 0.3, 2.3)] during weekdays [2.2 (95% CI: 1.2, 4.2)] vs weekend [0.6 min/day (95% CI: 0.3, 1.2)] and among those who were currently employed [10.3 (95% CI: 0.7, 3.1) vs not working [1.5 (95% CI: 5.3, 20.0)]. Those with greater than high school education accumulated 6.2 min/day more in MVPA than those with less than high school education but this difference was not statistically significant. LPA, MVPA and number of steps were significantly higher on weekdays compared to weekends. Sedentary time did not differ significantly between weekend and weekdays (Table 2). Pearson’s correlation showed a positive association between LPA and MVPA (r = 0.42, p < .001) indicating that as LPA increases so does MVPA. A negative correlation was observed between age and LPA (r = − 0.37, p = < 0.001).

**Table 2:** Mean daily minutes of sedentary time, LPA, MVPA and average daily step count.

	Sedentary			LPA			MVPA			Steps		
	Mean	95% CI		Mean	95% CI		Mean	95% CI		Mean	95% CI	
**All (Total N = 91)**												
	673.5	656.6	691	130.5	117.3	145.3	2.3	1.3	4.2	5030	4401	5749
**Season of monitoring (n)**												
Summer (38)	688.5	661.2	716.9	*153.2	132.5	177.2	*5.4	2.4	12.2	5818	4777	7086
Fall (8)	641.2	600.4	684.7	111.4	59	210.1	1.6	0.1	25.7	3929	2323	6646
Winter (35)	664.4	635.6	694.4	*111.9	94.2	132.9	*0.8	0.3	2.3	4637	3810	5643
Spring (10)	676.3	617.8	740.2	138.1	98.4	194	4.9	0.8	29.2	4757	2401	9424
**Days of the week**												
Weekdays	678.1	659.7	696.9	133.5	119.6	149.1	2.2	1.2	4.2	6217	5470	6964
Weekend	665.6	644.6	687.2	120.7	107.1	136	0.6	0.3	1.2	5264	4515	6014
**Sex**												
Women	694.6	671.8	718.3	124.6	106.2	146.3	1.8	0.8	4.5	4374	3613	5294
Men	657.4	633.6	682.1	135.3	116.7	157	2.8	1.2	6.4	5625	4672	6773
**Age (years)**												
20–59	688.8	654.6	724.7	*168.1	136.9	206.4	3.5	1.3	9.6	4847	3669	6404
60–79	671.5	650.8	692.9	*120.3	106	136.6	2.0	0.9	4.3	5109	4325	6036
≥80	628.1	544.8	724.1	106.4	55.8	203.2	2.2	0.0	117.2	4967	2880	8566
**BMI (kg m** ^**2**^ **)**												
18.5–24.9	685.1	638.5	735.1	*100.3	72.9	138	1.1	0.3	3.9	5025	3434	7352
25–29.9	661.2	637.5	685.7	*142.9	125.2	166.6	4.4	2.3	8.4	5491	4747	6451
≥30	688.4	660	718	133.4	110.5	154.1	1.1	0.3	3.9	4349	3174	5697
**Religion**												
Sikh	676.3	657.3	696	131.8	117	148.4	2.1	1.1	4.0	5152	4448	5969
Other	655.3	621.4	691.1	122.6	94.8	158.4	5.2	1.7	16.5	4304	3069	6035
**Marital status**												
Married	677.2	659.4	695.5	136	121.7	151.9	2.3	1.2	4.4	5106	4425	5891
Other	618.5	550.7	694.7	81.7	51.4	129.7	3.2	0.2	56.9	4490	2473	8153
**Years lived in Canada**												
<10	683.8	648.6	720.8	142.3	106	190.9	3.8	0.8	18.0	5052	3396	7515
≥10	668.6	649.1	688.6	128.5	113.6	145.3	2.3	1.2	4.6	5090	4371	5928
**Children under 12**												
None	659.7	639.8	680.2	125.9	109.4	144.9	2.3	1.1	5.1	4814	4037	5740
1 or more	697.7	664.3	732.8	139	116	166.6	2.5	0.9	7.3	5478	4383	6844
**Education**												
> High School	655.9	623.6	689.9	140.3	114	172.7	7.5	3.1	18.1	5490	4323	6972
< High School	668.5	638.6	699.9	114.4	91.9	142.4	1.3	0.4	4.0	4814	3705	6256
High School	691.8	663.9	720.9	144.1	127.4	163	1.9	0.7	5.2	4988	4039	6160
**Employment**												
Not working	672.2	652.8	692.1	123	109.8	137.7	1.5	5.3	20.0	4703	4017	5505
Working	673.1	634	714.6	157	118.1	208.8	10.3	0.7	3.1	6272	4768	8251
**Income**												
<$20,000	669	633.8	706.2	*96.5	76.2	122.4	0.8	0.2	4.1	4209	3183	5566
$20,000–$49,999	686.3	659.2	714.5	*139.0	117.9	163.9	2.5	1.0	6.2	5216	4245	6409
≥50,000	662.2	628.8	697.4	138	109.5	174	3.7	1.2	11.4	4924	3657	6628

Due to highly skewed time data, time in minutes was log transformed to calculate mean and then exponentiated.

LPA = Light physical activity

MVPA = Moderate-to-vigorous physical activity

* Groups significantly different from each other within the PA category at p = <0.05.

**Fig 1:**
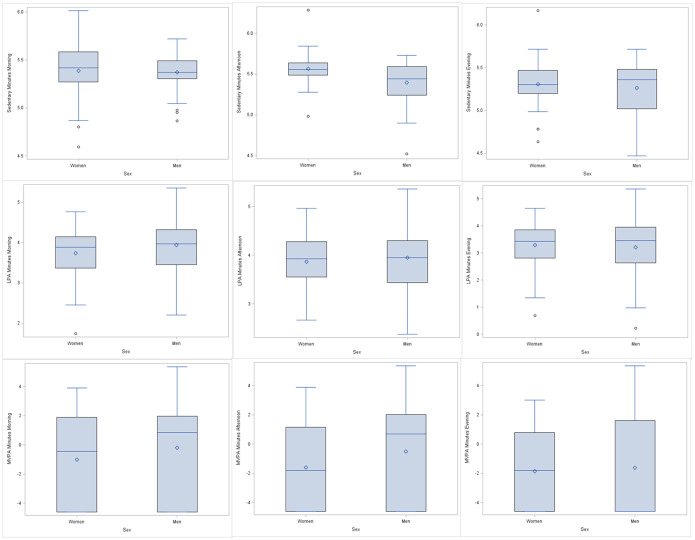
* Distribution of mean minutes per day of a) sedentary time and b) LPA and c) MVPA at various times of the day by sex (transformed data). *Repeated Measures ANOVA.

Accumulation of sedentary time and PA differed by time of day (Fig 1). When stratified by sex, an association was observed in women for sedentary time; women accumulated significantly more sedentary time in the afternoon than men [265.9 min (95% CI: 252.2, 280.4) vs 224.8 (95% CI: 212.3, 238)], (S1 Table).

Nineteen out of 91 participants (22%) met the PA guidelines of acquiring at least 150 minutes of MVPA/week.

### Correlations between wear time, select socio-demographic variables and physical activity outcomes

Wear time (average min/day on valid days) was significantly correlated with sedentary time (r = 0.65, p = <0.001), LPA (0.58, p = <0.001) and MVPA (r = 0.25, p = 0.02). Since higher mean minutes of sedentary time and various intensity PA could be because of a higher number of mean minutes of accelerometer wear time, we explored this association in sub-groups where significantly higher study outcomes corresponded with significantly higher mean minutes of wear time.

#### Season of monitoring

Table 2 shows mean minutes of both LPA and MVPA were significantly higher in summer (153.2 and 5.4 min/day) vs winter (111.9 and 0.8 min/day) respectively. Looking at the wear time distribution (S2 Table), we see that wear time was also significantly higher in summer (880 vs 803 min/day in winter). We found significant correlations between wear time and LPA for summer (r = 0.63, p = <0.001), and winter (r = 0.32, p = 0.02), which could explain higher levels LPA during these seasons. Similar correlations were not observed for MVPA thus, leading to the conclusion that higher levels of MVPA observed during summer were not due to a higher wear time during this season.

#### Days of the week

Mean minutes of accelerometer wear time were significantly higher for weekdays [850.4, (103)] vs weekend [820.2, (118.5)]. There was a medium strength correlation between wear time and LPA for weekdays (r = 0.51, p = <0.001) and a weak (but significant) correlation between wear time and MVPA during weekdays. Higher mean minutes accumulated over weekdays may thus be partly explained by higher mean wear minutes during weekdays vs weekend.

#### Age

Wear time was significantly different among all three age categories. LPA was significantly different among those between the ages of 20–59 (168.1 min/day) vs 60–79 years old (120.3 min/day) (p = 0.04) (Table 2). For LPA, significant correlations were observed between wear time and those in the age categories 20 to 59 years old (r = .68, p = <0.001 and 60 to 79 years of age (r = .47, p = <0.001). Higher minutes of LPA accumulated among 20 to 59-year-olds could thus be attributed to wear time. No significant correlations were observed between wear time and various age categories for MVPA.

#### Marital status

Married participants accumulated significantly higher mean minutes/day in LPA: (136.0) vs other (81.7) (p = 0.01) (Table 2). But this could be attributed to a significant correlation between mean min/day of wear time among married (r = 0.57, p = <0.001) vs other (r = 0.28, p = .54).

#### Income

For LPA, significant correlations were observed between wear time and all three categories of income; <$20,000 (r = 0.50, p = 0.02), $20,000 –$49,000 (r = 0.51, p = 0.001) and ≥ $50,000 (r = .67, p = < 0.001). Mean min/day of LPA were higher in those with an income between $20,000 –$49,000 (139.0 min/day) vs those with <$20,000 (96.5 min/day) (p = 0.03) (Table 2). Mean minutes of accelerometer wear time were also significantly higher in the medium income category [862, (SD 91.9)] compared to the lowest income category [790, (SD 83.1)] thus, high LPA in the medium income group could be partly explained by perhaps higher wear time in this category compared to < $20,000 income category (862 min/day vs 790 min/day).

#### Employment status

Mean min/day of MVPA as well as accelerometer wear time were significantly higher in those participants who were employed but no significant correlations were observed between wear time and MVPA. Thus, higher mean minutes of MVPA (10.3, 95%CI: 0.7, 3.1) in the working group cannot be attributed to higher mean minutes of accelerometer wear time.

### Socio-demographic characteristics associated with sedentary time and physical activity levels

Multivariable linear regression models for sedentary time, LPA and MVPA are shown in Table 3. For sedentary time and LPA, sex and BMI, explained 51% of the variability. After adjusting for wear time and BMI, there was an increase of 1.11min/day in sedentary time accumulated by women compared to men. A significant interaction effect between sex and BMI was observed for both sedentary time and LPA models. Women in the healthy and over-weight categories of BMI accumulated more sedentary time [742.5 min/day, (95% CI: 686.0, 803.6) and 691.3 min/day, (95% CI: 662.2, 721.6)], respectively than healthy [669.8 min/day, (95% CI: 637.7, 703.5)] and over-weight [645.7min/day, (95% CI: 624.8, 667.3)] men. Women in the obese category accumulated significantly fewer minutes in sedentary time [673.9 min/day, (95% CI: 646.2, 702.6) than obese men [700.5min/day, (95% CI: 660.3, 743.2)] (Table 3).

**Table 3:** Multivariable linear regression models for sedentary time, LPA and MVPA.

**Sedentary Time**						
	**Estimate** ^a^ **(ᵝ)**	**P**	**95% CI**		**R** ^**2**^	**P-Value**
					0.51	< .001
Intercept	336.97	< .001	284.29	399.41		
**Sex**						
Women	1.11	0.03	1.01	1.22		
Men (Ref)	0.00					
**BMI** (kg m^2^)						
≥30	1.04	0.25	-0.97	1.13		
25–29.9	-0.96	0.22	-0.90	1.02		
18.5–24.9 (Ref)	0.00					
**Wear minutes**	0.0008	< .001	0.0006	0.001		
**Interaction Sex & BMI**						
Women* BMI ≥30	-0.88	0.02	-0.77	-0.98		
Women * BMI 25–29.9	-0.96	0.52	-0.87	1.07		
Women *BMI 18.5–24.9	0					
Men* BMI ≥30	0					
Men * BMI 25–29.9	0					
Men *BMI 18.5–24.9	0					
**LPA**						
	**Estimate** ^b^ **(ᵝ)**	**P**	**95% CI**		**R** ^**2**^	**P-Value**
					0.51	< .001
**Intercept**	10.49	< .001	5.21	21.12		
**Sex**						
Women	-0.46	0.0002	-0.31	-0.69		
Men (Ref)	0					
**BMI** (kg m^2^)						
≥30	-0.90	0.5	-0.65	1.23		
25–29.9	1.20	0.14	-0.94	1.54		
18.5–24.9 (Ref)	0					
**Wear minutes**	0.003	< .001	0.002	0.004		
**Interaction Sex & BMI**						
Women* BMI ≥30	2.61	0.0003	1.58	4.26		
Women * BMI 25–29.9	1.86	0.008	1.19	2.92		
Women *BMI 18.5–24.9	0					
Men* BMI ≥30	0					
Men * BMI 25–29.9	0					
Men *BMI 18.5–24.9	0					
**MVPA**						
	**Estimate** ^c^ **(ᵝ)**	**P**	**95% CI**		**R** ^**2**^	**P-Value**
					0.22	0.002
**Intercept**	2.05	0.52	-0.23	18.41		
**BMI** (kg m^2^**)**						
≥30	0.84	0.92	-0.16	4.31		
25–29.9	2.84	0.18	-0.64	12.74		
18.5–24.9 (Ref)	0					
**Employment Status**						
Working	5.79	0.01	1.54	21.69		
Not working	0					
**Seasons**						
Summer	1.06	0.93	-0.16	7.59		
Fall	0.38	0.44	-0.03	4.67		
Winter	0.16	0.06	-0.02	1.08		
Spring	0					
**Multivariable exponentiated interaction effects models for sedentary time and LPA**						
**Sedentary Time Model**						
**Sex**	**BMI**	**Estimate**	**95% CI**			
Women	≥30	673.9	646.3	702.7		
	25–29.9	691.3	662.3	721.6		
	18.5–24.9	742.6	686.1	803.7		
Men	≥30	700.5	660.3	743.23		
	25–29.9	645.7	624.8	667.3		
	18.5–24.9	669.9	637.8	703.6		
**LPA Model**						
**Sex**	**BMI**	**Estimate**	**95% CI**			
Women	≥30	141.1	118.4	168.1		
	25–29.9	127.9	106.8	1531		
	18.5–24.9	59	42.4	82.3		
Men	≥30	113.8	88.8	145.7		
	25–29.9	152.6	132.9	175.1		
	18.5–24.9	126.9	103.3	155.9		

^a^ Exponentiated estimates pertain to differences in minutes of sedentary time

^b^ Exponentiated estimates pertain to differences in minutes of LPA

^c^ Exponentiated estimates pertain to differences in minutes of MVPA.

After adjusting for wear time and BMI, women accumulated 0.46 min/day lower time in LPA compared to men. Employment status and season were significantly associated with MVPA while wear time and BMI were not. However, including BMI improved the model fit and was therefore kept in the model. BMI, employment status and season of the year explained 23% of the variability in our model. Participants who were employed had higher MVPA (5.79 min/day) after adjusting for BMI and season they were assessed in.

## Discussion

To our knowledge, this is the largest study in North America that has directly measured PA and sedentary time and explored potential socio-demographic correlates of PA and sedentary time in South Asians. Overall, we observed high sedentary time and low MVPA. Female sex and BMI in the obese category (≥ 30) was associated with higher sedentary time and low LPA. BMI, employment status and season of the year explained the highest proportion of variability in MVPA with those who were employed having significantly higher mean minutes of MVPA after adjusting for BMI and season.

Study participants accumulated higher sedentary time than other studies with a South Asian sample. Compared to our participants, Castaneda-Gameros et al., reported 2 hours less sedentary time in older South Asian women [mean age 70.8 (8.1) years] in the UK [38]. However, this could be because of differences in wear-time; when sedentary time was expressed as percentage of wear time, ours is lower (62% vs 69% in Castaneda-Gameros study). Similarly, in another UK-based study, South Asian women [mean age 45.5 (10.0)] and men [mean age 48.9 (9.3) years] accumulated 2–2.5 hours less in sedentary time (measured via Actiheart) than in our current sample [41]. Using data from the National Health and Nutrition Examination Survey (NHANES), sedentary time was approximately 3 hours lower in the American adult population (464 min/day) compared to our sample despite similar mean wear time (852 min/day in NHANES vs. 839 min/day in our study) [33, 42]. Sedentary time was approximately an hour higher in our sample (673 min/day) compared to the mixed-ethnic cohort of adult participants in the Canadian Health Measures Survey (CHMS) (588 min/day) [43]. However, our comparisons with other studies must be interpreted with caution due to differences in methodologies, measurement device, cut-points and different socio-demographics of the populations being researched. For example, the majority of studies mentioned above used Actigraph accelerometers. One study comparing Actical and Actigraph accelerometers used thresholds developed specifically for each one and concluded that Actical estimated significantly less time spent in LPA (-16.3%), MPA (-2.8%), and VP (-0.4%) than the ActiGraph, but greater time spent sedentary (+20.5%) [44].

We observed higher sedentary time (689 min/day) in the younger age group (<59 years old) compared to those 60 and older. An absolute difference of 60 minutes of sedentary time between the youngest (20–59 years) age group (689 min/day) and the oldest (≥ 80 years) age group (628 min/day), while not statistically significant, is of concern as studies have reported a dose–response relationship between sitting time, all-cause mortality [45], cardio-metabolic biomarkers [46] and metabolic syndrome [47, 48]. Compared to those who spent less than 8 hours/day sitting, individuals who spent 8–11 hours/day Hazard Ratio (HR) 1.35; 95% CI 1.09–1.66) and more than 11 hours/day sitting (HR1.52; 95% CI 1.17–1.98) had a higher risk of all-cause mortality. For each hour/day spent sitting, there was an increase of 3% (HR 1.03; CI 95% 1.01–1.05) in the risk of all-cause mortality [45]. Health risks for South Asians appear to be even greater. A study on urban South Asian women observed these women being at higher risk of dysglycaemia at lower levels of sedentary behaviour and greater PA than western populations, indicating the need for re-visiting current PA guidelines for South Asians. According to this study, women viewing TV for >85 minutes were six times as likely to be dysglycaemic when compared to those spending less time viewing TV; 85% of people with dysglycaemia could attribute their condition to viewing TV >85 minutes a day after adjusting for other variables [48]. Higher sedentary time in the younger age category may be partly explained by competing demands such as work on relatively younger, first generation immigrants in our sample who may be still trying to establish themselves in their adopted country and may not have the option of taking time out for leisure time PA. Future mixed-methods research based on larger samples of younger South Asians need to investigate other factors that may be responsible for making this group relatively more sedentary.

On average, participants in our study took 5030 mean steps/day. Daily step counts have been inversely associated with BMI, hypertension, and diabetes [49]. According to one cross sectional study, individuals who took ≥5000 steps per day had a lower prevalence of metabolic syndrome than those who obtained fewer steps [49]. Another prospective cohort study on older adults concluded that a threshold of 4500 steps/day was found to best distinguish participants with the lowest risk of diabetes, where those taking ≥ 4500 steps/day, having 59% lower risk of diabetes, compared to those taking fewer steps (HR, 0.41, 95% CI, 0.25–0.66) [50]. According to Patel et al, adding even 2,000 more steps (about 1 mile) of walking above the 3,000 to 4,000 baseline steps each day is associated with lower all-cause mortality [51]. While the mean number of steps in our study (5030 steps/day) exceed the thresholds mentioned above, studies have recommended that PA guidelines be ethnicity-specific, providing evidence that South Asians need higher level of activity to confer a similar cardio-metabolic risk profile to adults of white European descent achieving current PA recommendations of 150 minutes per week [48, 52]. Thus, application of more stringent categories and classification system of Tudor-Locke and Bassett to this population makes sense. Under this classification, the majority of our study participants (46%) step count falls into the sedentary category, with 21% being classified as physically inactive, another 21% as moderately active; only 11% as physically active while 3% as very active [34]. Since steps have the advantage of being intuitive, easy to measure and objective [53] and since walking has often been cited as one of the most popular sources of PA for South Asian immigrants [54], encouraging this population to increase overall step-count, by providing activity monitors and setting step-count goals may help promote overall PA.

Our multivariable linear regression models revealed an interaction between sex and BMI for sedentary time and LPA. Women who were obese (BMI ≥ 30) were less sedentary than men in the same-category BMI, while women in the over-weight and healthy range of BMI were more sedentary than men in those categories. For LPA, women who were obese engaged in more LPA compared to men in the same-category BMI, while women in the over-weight and healthy categories of BMI accumulated fewer minutes in LPA compared to men in similar categories. In contrast, NHANES data shows increasing gradients and significant trends in men for sedentary time across BMI categories, but not for women [55]. Heinonen et al. in a cross-sectional study in a population-based sample of healthy adults, show leisure-time sedentary behaviour, primarily TV viewing time being directly associated with WC in both sexes, independent of the genetic obesity risk score, sleep duration, PA, energy intake and other covariates (p<0.002) [56]. Women in the obese category in our sample may have been more engaged in domestic activities such as cooking and cleaning around the house compared with men in the obese category and this may partly explain why they accumulated less sedentary time than men in a similar BMI category. Asking participants to keep activity diaries along with device-worn measurement of their PA and sedentary time would provide a fuller picture of not only the sources of sedentary time but also the contexts in which various intensity PA takes place.

While some prospective studies have shown a higher BMI leads to a more sedentary lifestyle in both men and women [57], a sedentary lifestyle has also been observed to cause a higher BMI [58, 59]. As a cross-sectional study, we were not able to examine causality. Evidence suggests that both mechanisms are at work on an individual level [60]. In one prospective, population based study, BMI, fat mass and WC predicted sedentary time but sedentary time did not seem to predict future obesity [60] while in another longitudinal study, higher values of all fat indices independently predicted longer sedentary time [61]. However, none of these studies showed an interaction with sex. Future studies would benefit from longitudinal study designs in order to delineate the direction of the observed link between BMI and sedentary time.

On average, women in our sample were significantly more sedentary than men. Examination of sex differences in sedentary time yields inconsistent results, with some population level studies like CHMS and NHANES reporting no sex differences in sedentary time [6, 42] while some other studies report men being more sedentary than women [62–65], and still others show women accumulating more sedentary time than men [66, 67]. The paucity of directly measured sedentary time data in South Asians limits an effective comparison of our study results with others. Few studies on South Asians have explored the sources of, or the reasons behind, such high levels of sedentary time observed in this population. Other than a universally recognized inability to recall sedentary time, some studies have indicated a lack of understanding regarding the concept of being ‘sedentary’ among South Asians—which often times, is associated with being ‘lazy’ and may therefore to some degree explain why self-reported sedentary time is low [16, 17, 68]. Other studies have highlighted complex interactions between social support, immigration, and culture—which creates barriers thereby lowering overall PA and increasing sedentary time in this population. These observations underscore the need to communicate not only the important role PA plays in promoting health but also knowledge regarding what constitutes sedentary behavior, its deleterious impact on health and how this could be replaced with LPA.

LPA is being increasingly investigated as a more feasible substitute of sedentary time than MVPA [69] with documented health benefits for chronic conditions [70]. Our sample’s observed LPA levels (130 min/day) are considerably lower than those reported by other studies that show LPA ranging from 207–574 min/day [37, 38, 41]. In our sample, 51% of the variability in LPA was explained by an association between BMI, sex, and wear time. BMI was also associated with LPA in a sample of South Asian women in the UK. [37]. In our sample, women in the overweight and healthy BMI categories had lower levels of LPA compared with men in those categories. This is contrary to what has been observed in previous studies where lower BMI was associated with higher LPA and lower sedentary time in older adults [71, 72]. High sedentary time and low LPA among women in our sample could partly be explained by the unique family structure of South Asians who tend to live with extended family [73]. The ‘well-meaning, benevolent protection’ [74] shown towards the elders by the younger members of the household may discourage them from doing simple tasks around the house, thereby, reducing overall PA. These findings however warrant further investigation in studies based on qualitative research.

MVPA in our sample (mean 2.3 min/day) was very low in comparison with CHMS 2012–13 data (mean15 min/day) [43]. Moreover, unlike CHMS and NHANES there were no significant age-related differences in MVPA within our sample. This could be because we had a very small sample of those in the 20 to 59 age range (24%). Our multivariable models showed that employment status, season and BMI were the strongest predictors of MVPA. Employment status has been associated with overall sedentary time and PA. Adults who work a full day spend ∼31% of their day on the job, therefore the contribution of work-related PA or inactivity to total PA and inactivity can be substantial [75]. In the US, unemployed men and women were less active than their employed counterparts (Hagstromer 2010). One study examining the relationship between employment status and directly measured PA and sedentary time separately for men and women in US and Sweden, concluded significant differences in activity levels and sedentary time between employed and unemployed respondents in US but not in Sweden [76]. Men were observed to be less active and more sedentary when unemployed, women were also less active when unemployed, but the opposite pattern was observed for sedentary time. This could be because unlike unemployed men, unemployed women tend to replace work with active pursuits or LPA, such as domestic activities. However due to the small number of working women in our sample (n = 3), we were not able to explore this association by sex in our study.

PA and sedentary time have been associated with the type of occupation and often differ in the expected direction for active and sedentary occupations where those involved in active occupations having higher cpm and less sedentary time than those with sedentary occupations [76]. Few device-measured studies on South Asians have explored the association of employment on sedentary time and PA thus limiting our ability for a comparison with a similar population. More specific information regarding the kind of employment our participants were engaged in would have enabled us to better understand the source of higher mean levels of sedentary time/MVPA. We speculate that the majority of our working sample (which was predominantly male) may have been engaged in activity intensive occupations which may, to some extent, explain higher levels of MVPA. Future studies should further explore this possible association.

Participants in our study accumulated higher minutes in MVPA during summer than in fall. A systematic review exploring the effect of season and weather on PA concluded that levels of PA vary with seasonality, and that the ensuing effect of poor or extreme weather can act as a barrier to participation in PA among various populations thus decreasing PA levels [77]. Qualitative research has identified weather as one of the most common barriers to a physically active lifestyle among South Asian immigrants in western countries [54, 78]. Our multivariable models show a significant association between MVPA and season with highest MVPA accumulated in the summer months. A focus on outdoor PA interventions during the summer and indoor during the winter may increase uptake of PA in this population.

Only 22% of our sample met the PA guidelines of achieving 150 minutes of MVPA over at least 5 days a week. However, this may be an overestimate as we included all those participants who had three to six valid days, and multiplied their average daily MVPA by seven to obtain a weekly sum. The actual proportion my thus be even lower. This is consistent with other studies that compared PA of South Asians with other ethnic groups, [41, 79]. This is of concern as South Asians may need to undertake even greater levels of MVPA (close to 266 min/week) than Europeans to mitigate their elevated risk for metabolic syndrome and exhibit a similar cardio-metabolic risk profile [52, 80].

### Strengths and limitations

This study is the largest in North America that is based on device-worn direct measures of PA and sedentary time in the South Asian ethnic group and the first in this region to explore correlates of PA and sedentary time in a group at high risk for type 2 diabetes. Compliance to wearing the accelerometer was excellent with 91% of the sample providing at least three days of valid data. Moreover, this study provides insight into patterns of PA and sedentary time of South Asian men and women, and identifies some of the modifiable factors that can be addressed to reduce health disparities related to high-risk ethnic groups. These findings are a significant contribution to literature and have important implications for health promotion practice.

The study, however, is not without its limitations. This was a convenience sample recruited primarily from Gurdwaras. The non-random nature of our sample as well as the fact that it was recruited from religious places of worship may have introduced selection bias. Moreover, of the various South Asian sub-groups, only one sub-group (comprising of Punjabi Sikhs), was heavily represented in our sample (90%). Other studies have documented significant South Asian sub-group differences regarding PA levels and sedentary time and associated risk of chronic disease [11]. While our goal was a diverse sample representing a wide spectrum of age, education and employment status, the majority of participants were older and retired. All these factors limit generalizability to the larger South Asian community living in Canada and makes comparison with other studies difficult. Also, we did not investigate potential confounders like psychosocial stress, depression and cultural factors that are known to affect PA and sedentary behavior [81, 82]. Additionally, there are inherent limitations in the accelerometer device as it does not measure the added energy expenditure associated with upper body movement (for example, weight-lifting, shoveling snow and other upper body movement), load carrying, or walking up an incline [6]. Omnidirectional accelerometers such as MiniMitter and Actical, while they assess acceleration in multiple directions, are most sensitive to movement in the vertical plane. This may potentially lead to an overestimation of sedentary time and underestimation of LPA [6]. This is particularly relevant for older women, many of whom are usually engaged in light housework around the house. There is limited published literature regarding adult cut-points for the Actical. While we applied the same cut-points as the CHMS [6, 34]. These cut-points were derived from a lab-based validation study with non-South Asian adults and their use within other populations has not been tested [35, 83]. Lastly, as this was a cross-sectional study our findings are descriptive and do not establish a causal link between socio-demographic characteristics and PA outcomes. Nevertheless, our study makes important contribution to the field of directly assessed sedentary time and PA and emphasizes the need for future research based on longitudinal design.

## Conclusion

High sedentary time, and low LPA in a high-risk population indicate the need to actively involve this community in a dialogue about detrimental health effects of a sedentary lifestyle. Health promotion efforts need to be focused on shifting sedentary time into LPA while trying to improve overall MVPA levels in this population. Despite being at a high-risk for type 2 diabetes, PA levels remain low in this group. However, many of the study participants were not aware of their high-risk status till they underwent screening during recruitment. This highlights a missed opportunity in health service delivery for early detection of high diabetes risk and targeted PA promotion messages for this group so as to delay the onset of Type 2 diabetes. A regular screening for diabetes risk and assessment or prescription of PA as part of routine care by a family doctor could be an effective mechanism and increase PA levels thus generating positive clinical outcomes.

Moreover, population level surveys like the CHMS in Canada and NHANES in the US need to include nationally representative samples of South Asian people that have been identified at a high risk for chronic disease. In the meantime, we need methodologically strong studies based on randomly selected samples so findings can be generalized to the South Asian population at large. Furthermore, due to a considerable heterogeneity within South Asians, future studies also need to investigate sub-group differences to ensure development of effective, culturally sensitive public health interventions targeting all sub-groups.

## Supporting information

S1 Table Distribution of mean minutes per day of sedentary time, LPA and MVPA at various times of the day by sex (transformed data). (DOCX)

S2 Table Mean minutes of accelerometer wear time by sub-groups. (DOCX)
