# Supplementary material for: Device-worn measures of sedentary time and physical activity in South Asian adults at high risk for type 2 diabetes in Metro-Vancouver, Canada
Source: PLoS One. 2022 May 5;17(5):e0266599. doi: 10.1371/journal.pone.0266599 (PMC9070884; doi:10.1371/journal.pone.0266599)
Supplement: S1 Table — (DOCX) [file pone.0266599.s001.docx]

S1 Table: Distribution of mean minutes per day of sedentary time, LPA and MVPA at various times of the day by sex (transformed data).

| **Time of Day** | **PA level** | **Sex** | **Mean Min per day** | **P-Value** |
| --- | --- | --- | --- | --- |
| **Morning** |  |  |  | 0.93 |
|  | Sedentary |  |  |  |
|  |  | Women | 216.70 |  |
|  |  | Men | 215.80 |  |
|  | LPA |  |  | 0.26 |
|  |  | Women | 42.70 |  |
|  |  | Men | 49.50 |  |
|  | MVPA |  |  | 0.25 |
|  |  | Women | 0.41 |  |
|  |  | Men | 0.84 |  |
| **Afternoon** |  |  |  | <.0001 |
|  | Sedentary | Women | 265.90 |  |
|  |  | Men | 224.80 |  |
|  | LPA |  |  | 0.66 |
|  |  | Women | 48.30 |  |
|  |  | Men | 51.00 |  |
|  | MVPA |  |  | 0.14 |
|  |  | Women | 0.25 |  |
|  |  | Men | 0.57 |  |
| **Evening** |  |  |  | 0.41 |
|  | Sedentary | Women | 203.40 |  |
|  |  | Men | 193.90 |  |
|  | LPA |  |  | 0.35 |
|  |  | Women | 28.90 |  |
|  |  | Men | 24.40 |  |
|  | MVPA |  |  | 0.80 |
|  |  | Women | 0.16 |  |
|  |  | Men | 0.19 |  |
|  |  |  |  |  |
