# Supplementary material for: Device-worn measures of sedentary time and physical activity in South Asian adults at high risk for type 2 diabetes in Metro-Vancouver, Canada
Source: PLoS One. 2022 May 5;17(5):e0266599. doi: 10.1371/journal.pone.0266599 (PMC9070884; doi:10.1371/journal.pone.0266599)
Supplement: S2 Table — (DOCX) [file pone.0266599.s002.docx]

S2 Table: Mean minutes of accelerometer wear time by sub-groups.

|  | **N** | **Mean** | **SD** | **Minimum** | **Maximum** | **95% CL Mean** | | **P-value** |
| --- | --- | --- | --- | --- | --- | --- | --- | --- |
| **Total** | 91 | 839.3 | 97.5 | 612.4 | 1044.2 |  |  |  |
| **Weekdays** |  | 850.4 | 103.6 | 617 | 1066.8 |  |  | 0.002 |
| **Weekend** |  | 820.2 | 118.5 | 601 | 1125 |  |  |  |
| **Sex** |  |  |  |  |  |  |  |  |
| Women | 40 | 848.3 | 80.1 | 685.6 | 1018.8 | 822.7 | 873.9 | 0.42 |
| Men | 51 | 832.2 | 109.6 | 612.4 | 1044.2 | 801.4 | 863 |  |
| **Marital Status** |  |  |  |  |  |  |  |  |
| Married | 82 | 848 | 95 | 663 | 1044.2 | 827.1 | 868.9 | 0.002 |
| Other | 7 | 732 | 76.7 | 612.4 | 826.8 | 661.1 | 803 |  |
| **Children under 12** |  |  |  |  |  |  |  |  |
| 0 | 60 | 822.5 | 97.4 | 612.4 | 1018.8 | 797.4 | 847.7 | 0.036 |
| 1 or more | 28 | 869.5 | 93.5 | 685 | 1044.2 | 833.3 | 905.8 |  |
|  |  |  |  |  |  |  |  |  |
| **Employment** |  |  |  |  |  |  |  |  |
| Not working | 67 | 823.6 | 90.2 | 612.4 | 1018.8 | 801.6 | 845.6 | 0.01 |
| Currently working | 22 | 885.4 | 109.8 | 663.3 | 1044.2 | 836.7 | 934.1 |  |
| **Age** (categories) |  |  |  |  |  |  |  |  |
| 20-59 | 24 | 894.1* | 85.8 |  |  |  |  | 0.002 |
| 60-79 | 62 | 824.4*ᶨ | 93.3 |  |  |  |  |  |
| 80> | 5 | 760.9*ᶨ | 98.3 |  |  |  |  |  |
| **BMI** (categories) |  |  |  |  |  |  |  |  |
| 18.5 - 24.9 | 18 | 831.7 | 96.2 |  |  |  |  | 0.912 |
| 25 - 29.9 | 46 | 839.1 | 101.2 |  |  |  |  |  |
| ≥30 | 27 | 844.6 | 95.3 |  |  |  |  |  |
| **Education** |  |  |  |  |  |  |  |  |
| < High School | 25 | 839.7 | 104.9 |  |  |  |  | 0.411 |
| High School | 35 | 823.6 | 107.9 |  |  |  |  |  |
| > High School | 29 | 856.7 | 79.2 |  |  |  |  |  |
| **Income** |  |  |  |  |  |  |  |  |
| <20,000 | 19 | 790.0** | 83.1 |  |  |  |  | 0.03 |
| 20,000 - 49,999 | 38 | 862.0** | 91.9 |  |  |  |  |  |
| >=50,000 | 24 | 837.4 | 105.9 |  |  |  |  |  |
|  |  |  |  |  |  |  |  |  |

** Age category 1 is significantly different from category 2 & 3*

*ᶨ Age category 2 is significantly different from category 3*

*** Income category 1 is significantly different from category 2*
